# Navigating the Grey Zone: The Impact of Legislative Frameworks in North America and Europe on Adolescent Cannabis Use—A Systematic Review

**DOI:** 10.3390/bs14060484

**Published:** 2024-06-06

**Authors:** Barbara Jablonska, Lilian Negura

**Affiliations:** School of Social Work, Faculty of Social Sciences, University of Ottawa, 120, University Private, Ottawa, ON K1N 6N5, Canada; bjabl052@uottawa.ca

**Keywords:** adolescent cannabis use, legislative impact, policy changes, North American and European comparison, societal attitudes, demographic influences

## Abstract

Objectives: This paper aims to systematically review the impact of legislative framework changes in North America and Europe on adolescent cannabis use. It not only seeks to examine the prevalence of adolescent marijuana use following legislative changes but also to identify the driving forces behind fluctuations in use and to address the gaps left by previous studies. Methods: A systematic literature review was conducted in selected databases. After screening English-language publications dating from 2013 to 2023 (*n* = 453 studies), 24 met the inclusion criteria. Articles were considered if they analyzed the impact of legislative changes on adolescent cannabis use in countries across North America and Europe. Synthesis: The overall findings suggest an inconsistency regarding the prevalence of cannabis use among youth and adolescents following policy changes. The effects of modifications in cannabis policies on marijuana consumption are complex and influenced by various factors. These include the details of legislation, societal perspectives, enforcement methods, socioeconomic status, and cultural background. Conclusions: The results of this analysis reveal a nuanced reality. Although research suggests a rise in cannabis use after legalization, there are variations in the outcomes observed. This highlights the significance of considering context and demographics. Moreover, studies shed light on how specific policy changes, such as depenalization, can affect cannabis use.

## 1. Introduction

The changing landscape of the law in different parts of North America and Europe presents a significant opportunity to understand how legal changes affect the use of cannabis among adolescents, which is a matter of paramount importance for public health.

In 2018, Canada legalized the use of recreational cannabis. This change aimed to address the perceived deficiencies of the restrictive control system while aligning with the wider Western trend of modifying cannabis regulations [1]. In the United States, both medical marijuana laws (MML) and recreational marijuana laws (RML) have undergone changes. In 1996, California led the way by becoming the first state to legalize medical marijuana. Despite federal restrictions on cannabis, subnational entities have taken diverse approaches to cannabis policies based on their powers granted by the Tenth Amendment and direct democratic mechanisms. As a result, significant reforms have occurred at the state level, with cannabis approved in 37 states and non-medical cannabis regulated in 18 states [1].

As North America continues to evolve its cannabis policies, Europe also presents a varied and complex legal landscape regarding cannabis regulation. In Europe, cannabis policies vary from country to country. The European Union (EU) has no unified position on this issue, and instead tries to adapt its legal framework to national variations. This approach aims to strike a balance between on the one hand standardization and on the other hand granting each member state some independence in determining their cannabis policies [2]. Despite these variations, all EU member states have implemented measures aimed at reducing the harm associated with drug use [1]. While some countries such as Poland, Germany, and the Netherlands allow for medical cannabis use, the specific policies regarding its use may differ [3,4]. There is also a range of approaches when it comes to policies that govern cannabis use. In the Netherlands, there is a regulated distribution of cannabis, whereas in countries such as Hungary, possession of cannabis can lead to imprisonment due to the laws in place [5]. The Netherlands’ unique political architecture, characterized by consociational democracy and proportional representation, facilitated a shift toward a liberal, expertise-led approach to cannabis policy [1].

Health professionals often express heightened concerns about adolescents in relation to substance use and the policies surrounding it. Among 15- to 24-year-olds, substance use stands as the primary modifiable risk factor for global morbidity; specifically, cannabis dependence contributes more significantly to morbidity among adolescents worldwide than any other illicit drug [6]. Moreover, the evolving landscape of cannabis legalization in various regions poses a unique challenge in navigating policy, as the shift in legality has prompted debates about its accessibility and potential impact on adolescent patterns of use [7]. Therefore, balancing the need for effective intervention strategies with the societal shifts in perception remains a crucial task for policymakers and healthcare professionals alike for sustainable development in society.

The majority of previous systematic reviews done worldwide on the prevalence of cannabis use among youth focused on one country or geographical area, with the majority of the studies being done in the USA. It is therefore necessary to conduct a systematic review of studies on cannabis use by young people in countries with diverse legislative approaches to regulating cannabis use. North American and European countries, while having similar cultural, social, and politico-economic contexts, are nevertheless characterized by different approaches to cannabis use. In addition, a critical number of studies on cannabis use by young people have been carried out in some of these countries, making it possible to conduct a systematic review about North America and Europe. Therefore, our main research question for this review is: What is the effect of legislative framework changes in North American and European countries on adolescent cannabis use? To the best of our knowledge, comprehensive reviews addressing this subject across both continents simultaneously, particularly among countries with more lenient marijuana laws (excluding legalization), have been insufficient to date.

This review aims to examine not only the prevalence of adolescent marijuana use but also the driving forces behind the fluctuations in use. By doing so, the review seeks to establish a global understanding of marijuana use among young people following legislative changes on cannabis use. Furthermore, this review serves as a valuable resource at a time when societal perspectives on cannabis have been evolving and legalization efforts have been expanding, and when there is a need for nuanced strategies to address the impact of cannabis use on adolescent health and to build sustainability in various domains including society and education.

## 2. Methods

This study is a systematic review, carried out according to the PRISMA guidelines. The review was conducted using five databases: PubMed, JSTOR, APA, PsycNet, and SCOPUS. The selection of the databases took into account easy access and wide coverage of reliable journals. We looked for articles that focused on patterns of youth cannabis use after the legislative changes from January 2014 until July 2023. As cannabis regulations have changed rapidly, the search was limited to the last 9 years to focus not only on the most recent findings but also on the historical changes to cannabis regulation.

### 2.1. Eligibility Criteria

The main criterion for selecting studies for this research was to investigate the relationship between changes in cannabis policy and cannabis use among adolescents.

Inclusion Criteria:

- All studies involving participants under the age of 25. The age limit was set at 25 due to varying legal adulthood ages across countries, ranging from 18 to 21. Studies commonly sample participants within wide age ranges like 12–25 or 18–24 without differentiating between specific ages. Recognizing this variability, the authors deemed it necessary to include studies up to the age of 25 to ensure comprehensive coverage and account for differences in legal adulthood thresholds worldwide.
- There were no limitations based on the nationality or gender of participants; thus, studies from all countries in North America and Europe, and participants of any gender, were considered.
- Articles that focused solely on or specifically distinguished marijuana use.

Exclusion Criteria:

- Articles focusing on adults—participants older than 25.
- Articles written in languages other than English. English was selected as it is the common language for both authors to ensure mutual understanding and effective collaboration.
- Articles that focused on the polyuse of substances and did not specifically distinguish marijuana in the results.
- Articles that focused on substances other than marijuana.

#### 2.1.1. Selection Criteria

**Figure 1:**
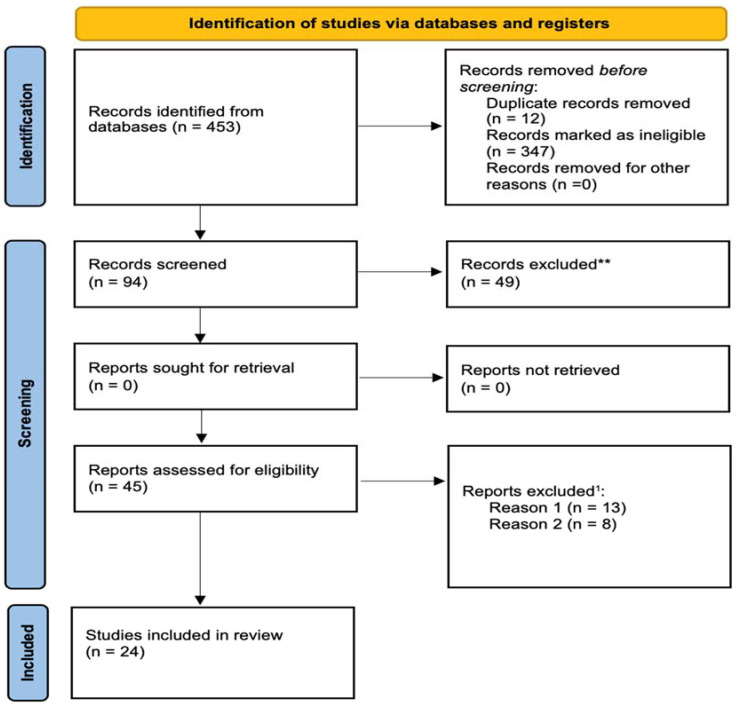
PRISMA flow diagram showing the selection of studies on the impact of legislative frameworks in North America and Europe on adolescent cannabis use. ^1^ At this stage reports have been excluded for two reasons: firstly, the absence of distinction between adults and youth (reason 1), and secondly, the lack of substance differentiation in cases of poly-use (reason 2). ** The records either lacked data (theoretical articles) or were irrelevant to the subject.

This study included only full-text original peer-reviewed articles written in English and focusing on observation and intervention studies. Articles excluded were systematic or narrative reviews, analyses not specific to adolescents, non-English articles, and those discussing polysubstance use without specifying substances. The searched keywords were “Impac* AND drug legislation OR marijuana legislation AND adolesc* OR youth* AND marijuana OR drug AND use”, “Effect* AND drug policy OR cannabis regulation AND teenage* OR youth* AND marijuana OR substance AND consumption”, “Influence* AND legal framework for drugs OR legislative measures on marijuana AND adolescent* OR youth* AND cannabis OR substance use”. After screening titles and abstracts, 408 articles were excluded, leaving 45 for further evaluation. Each of these articles underwent an assessment to determine if they met the inclusion criteria and objectives of the study. As a result, we included a total of 24 articles. The main reasons for excluding articles were that the participants fell outside the target age range (above 25 years) or that their content did not align with the study’s objectives. Figure 1 shows the process followed to select articles for this review.

#### 2.1.2. Data Extraction Tool

A content analysis informed by the work of Negura [8] and Braun and Clarke [9] was carried out using NVivo 1.7.1 for MAC. First, the results relevant to our research object, i.e., the policy changes and young people’s cannabis use, were identified. These outcomes were coded according to the type of legislative change, such as legalization, decriminalization, or variations in penalties. We then classified the different cannabis-related behaviors of young people (increased or decreased consumption, etc.) according to these types of legislative change. We also classified the different explanations favored by the various authors to explain these results. The articles identified were evaluated independently by the two co-authors to ensure consistency.

Criteria for Coding and Categorizing Outcomes:

- Participant Characteristics:
  Information regarding participants’ age, gender, nationality, and any relevant demographic factors was systematically coded and categorized to understand the demographic profile of the study populations. Codes included age, gender, nationality, socioeconomic status, and other pertinent demographic variables.
- Cannabis Policy Changes:
  Any alterations in cannabis policies within the studied regions were meticulously and comprehensively coded and categorized. This encompassed the nature of legalization (medical vs. recreational), types of restrictions imposed, and amendments to existing laws and enforcement measures (e.g., reduction of the severity of the penalties). Codes included the type of policy change (medical/recreational legalization, reduction of the severity of the penalties, or increase of the penalties), restrictions (e.g., age limits), and specific legal amendments.
- Impact of Policy Changes on Cannabis Use:
  The effects of legislative framework changes on adolescent cannabis use were analyzed in depth. This involved examining shifts in prevalence rates and alterations in patterns of use (e.g., frequency and consumption methods) and identifying any observed trends or correlations. Codes included increase in use, decrease in use, no change in use, changes in consumption patterns, and any emerging trends or correlations observed in the data.

Given the descriptive and narrative nature of the literature review we carried out, the heterogeneity of the results was ensured according to the different use patterns and types of legislative change. Our study did not set out to assess the bias and quality of the data presented.

#### 2.1.3. Data

Table 1 provides an overview of all 24 articles. The majority of these studies (12 articles) were conducted in the United States of America (USA) while seven were from Canada and six used data from the following countries: Bulgaria, Croatia, the Czech Republic, Denmark, Estonia, Finland, France, Greece, Hungary, Iceland, Italy, Latvia, Lithuania, Malta, the Netherlands, Norway, Poland, Portugal, Romania, the Slovak Republic, Slovenia, Sweden, Ukraine, and the United Kingdom.

The studies’ data were predominantly sourced from various reliable channels, including official surveys, national databases, and well-organized research surveys. The sample sizes across the studies varied significantly, ranging from 281 participants (minimum) to 1,179,372 participants (maximum). There was substantial heterogeneity in the measurement of outcomes and in the analytical techniques employed. Consequently, the data are presented descriptively.

## 3. Synthesis

This exploration of adolescent cannabis consumption in relation to changes in drug policies presents a nuanced picture, as depicted in Table 1. Research suggests that the legalization of cannabis (RML) in the United States has been associated with an increase in cannabis use among college students aged 18–26 [9]. The broader impact of changes on use becomes apparent also from the findings of Bailey et al. [19], who link non-medical cannabis legalization in the U.S. to a rise in self-reported cannabis use among young individuals. Similarly, Zuckermann et al. [23] discovered a rise in cannabis use among students in Canada, particularly during discussions surrounding the legalization of cannabis. Fischer et al. [12] observed also an increase in use among young adults aged 18–24 following the implementation of recreational cannabis laws.

However, Harpin et al. [11] found no change in cannabis use among students from grades 6 to 12 in Colorado after RML was implemented, highlighting the influence of pre-legalization initiation and local attitudes on consumption patterns. Rotermann’s [7] research, in turn, indicated that policy changes and societal acceptance have had an influence on cannabis use patterns. Notably, there was a decrease in cannabis consumption among 15–17-year-olds until 2017 and an increase among 18–24-year-olds from 2018 to 2019. Similarly, studies such as Wang et al.’s [15] discuss rates of cannabis use among college students residing in states with RML, indicating age- and gender-specific effects resulting from changes in cannabis policies. Cerdá et al. [20] also report effects across age groups, with a decrease observed among 8th graders and no significant change among 12th graders following medical cannabis legalization.

A study conducted by Benedetti et al. [5] in Europe demonstrates that specific policy changes, such as depenalization, can have specific effects on cannabis use. Significant changes in the prevalence of use are associated with two policy shifts: an increase in use is observed after the relaxation of penalties through the dropping of minor charges while stricter non-prison penalties lead to a decrease in use.

However, it is essential to acknowledge that the studies under review here utilize a range of methodologies, including longitudinal surveys and cross-sectional analyses. The diversity in sampling methods, such as convenience sampling, random sampling, or school-based surveys, can impact the representativeness of the study samples. Moreover, discrepancies in data collection tools, such as self-reported surveys, administrative records, or interviews, can introduce variations in the measurement of cannabis use behaviors and policy-related variables.

The geographical context also plays a crucial role, with studies conducted in different regions being influenced by unique cultural, social, and legal contexts that shape adolescents’ attitudes toward cannabis and their behaviors. Furthermore, differences in the timing of policy implementation across regions can lead to variations in the observed effects on cannabis consumption due to differences in adolescents’ exposure to policy changes.

Both researchers and policymakers should approach these findings cautiously, recognizing the limitations imposed by methodological variability. Attempting to generalize results across studies or regions may oversimplify the intricate relationship between drug policies and teenage cannabis consumption.

To summarize, the relationship between changes in drug policies and teenage cannabis consumption is complex and is influenced by factors such as local attitudes, age, and gender. In the next section, we will explore data on how young people’s cannabis use relates to policy changes.

### 3.1. Prevalence According to Policy Changes

As shown in Table 2, the findings of 20 articles revealed an increase in youth cannabis use following policy changes, while five articles reported a decrease. Seven additional articles suggested no noticeable change in use among young people (The total number of articles does not add up to 24, as certain articles report multiple behaviors contingent on the participants’ age bracket).

#### 3.1.1. Increased Use of Cannabis

##### Recreational Legalization

The data collected reveal a pattern of increased cannabis use among young people after the legalization of recreational cannabis in the United States and in Canada. Various studies conducted by Bae and Kerr [10], Rusby et al. [18], Bailey et al. [19], Zuckerman et al. [23], Evans et al. [24], and Stormshak et al. [28] in the USA, as well as Fischer et al. [12], Doggett et al. [21], and Mennis et al. [26] in Canada, all support this finding. These researchers suggest that several factors contribute to this rise, including a decrease in perceived risks associated with using cannabis, the easier availability of the drug, and its increasing acceptance within society.

##### Depenalization

The recent research conducted by Mæland and colleagues [13] sheds light on the following trend: It revealed that as people grow older, there seems to be a rise in cannabis consumption within families with lower incomes. This suggests that socioeconomic factors play a role in influencing the use of cannabis among individuals.

##### Quebec’s Legal Age Adjustment

The study conducted by Nguyen and Mital [14] adds a perspective to the context by examining the impact of raising the legal age for cannabis sales in Quebec. In comparison to provinces that did not implement this type of legal adjustment, the findings indicate that there was a decrease in cannabis use among 18- to 20-year-olds. This suggests that age-specific laws can effectively reduce cannabis consumption within the intended age brackets.

#### 3.1.2. No Significant Change in Cannabis Use

##### Recreational Cannabis Legalization

Although there has been an increase in cannabis use after it was legalized, studies conducted in Colorado (USA) by Harpin et al. [11], in the USA as a whole by Doran et al. [22], and in Canada by Nguyen et al. [27] did not observe significant changes in cannabis use among young individuals. This could be attributed to factors such as the prevalent use of cannabis before legalization, effective public-health campaigns, or specific local circumstances that mitigated the impact of legalization. The authors argued that while the legalization of cannabis made it more accessible to people, it also heightened their perception of its adverse effects.

##### Medical Cannabis Legalization

According to US data [16], there was no significant correlation found between youth who resided in states where medical cannabis was legalized and their recent cannabis use. Nonetheless, young individuals in these states were noticeably more likely to have begun using cannabis within the previous 12 months.

#### 3.1.3. Mixed Effects

##### Cannabis Legalization

According to the research conducted by Mauro et al. [16] and Cerdá et al. [20], the impact of legalizing cannabis in the United States on cannabis use by young individuals was found to be varied. While certain groups did not experience any alterations in their cannabis consumption, others reported an increase. This discrepancy may arise from variations in state regulations regarding the accessibility of cannabis and from differences in enforcement levels.

##### Decriminalization and Depenalization

In 20 European nations, decriminalization and depenalization of cannabis have the potential to increase the use of the drug among youth, according to a study by Benedetti et al. [5]. This may be explained by factors such as less severe legal repercussions changing public perceptions and perhaps even a growing belief that cannabis is generally safe. However, the study also shows a correlation between a rise in non-incarceration punishments and a decrease in cannabis use.

##### Penalty Reduction/Increase

The study carried out by Wieczorek et al. [4] revealed a noteworthy rise in cannabis experimentation among Polish adolescents following a reduction in severity of penalties for cannabis use. Except for the Czech Republic, this rate was higher than those observed in several other European countries, where a fall in use was more common. Conversely, Vaičiūnas et al. [29] found that across ten years in the Baltic states and Poland, cannabis consumption among young males varied more than among young females. Smyth et al. [6] discovered that variations in punishments were consistent with the deterrence hypothesis in several European nations, where more lenient penalties led to an increase in teenage use and where tougher penalties led to a drop in use.

## 4. Discussion

Changes in cannabis policy in recent years have raised public health concerns. Studies show that adolescents’ cannabis use behavior presents a complex picture as a result of these changes. Understanding this behavior is essential to developing public policies that promote the sustainable development of society.

Evidence of increased cannabis use among young people following the legalization of cannabis in countries such as the USA and Canada raises questions about its public health implications, given the long-term effects of cannabis on adolescent health [30], particularly on brain development [31]. Policy-makers need to balance the benefits of legalization, such as controlling distribution [32] and reducing costs to the justice system [33], against the risks of a potential increase in consumption among young people. Powerful public health campaigns to raise awareness among young people of the dangers of cannabis use must necessarily complement the implementation of cannabis legalization policies [34].

Evidence of increased cannabis use among older young people from lower socioeconomic backgrounds underlines the importance of targeting poverty reduction among young people to prevent their use. Policies must take into account not only the legal aspects of cannabis use, but also the social determinants of health [35]. Incorporating measures into cannabis policies that strengthen the social welfare system, education, and economic opportunities of vulnerable young people could offset these negative consequences [36].

For instance, adolescents from lower socioeconomic backgrounds may face increased exposure to environments where cannabis use is prevalent, such as neighborhoods with higher rates of substance use or limited access to recreational activities. Additionally, economic instability and lack of educational opportunities can contribute to feelings of hopelessness and lead some adolescents to turn to substance use as a coping mechanism.

Furthermore, the enforcement of cannabis policies may disproportionately affect marginalized communities, exacerbating existing social inequalities. For example, strict enforcement measures in low-income neighborhoods may result in higher arrest rates for cannabis-related offenses among adolescents from these communities, leading to long-term consequences such as criminal records and barriers to employment and education.

Evidence of the effectiveness of age-based cannabis legislation, as implemented in Quebec [14], demonstrates the potential of youth-specific policies. Establishing an adjusted legal age for the purchase of cannabis products can help shape the consumption behavior of young consumers [37]. This practice could align these new cannabis policies with the sustainable development needs of societies.

The absence of conclusive data on the effect of legalizing the medicinal use of cannabis on young people’s cannabis consumption, and of data on the growth in the number of young first-time users following this policy change, allows us to affirm, on the one hand, the fairly effective differentiation of medical and recreational use [38], and on the other hand, the importance of taking measures against non-medical use by young people.

According to studies carried out in Europe, decriminalization and depenalization policies have had varied and complex effects on young people’s use of cannabis. This reveals a subtle effect on young people’s consumption behavior of social representations [39], which must be taken into account when developing these policies. Policy-makers need to take into account the representational dynamics associated with the implementation of these policies, dynamics which may thus contribute to an increase in cannabis consumption among young people [40].

Despite the limited number of studies, a nuanced and cautious analysis reveals that more open legalization or decriminalization policies do not necessarily lead to increased marijuana consumption among adolescents and young adults. Harpin et al. [11] found no significant increase in adolescent use or changes in risk perceptions following Colorado’s legalization, though there was an increased perception of easy access. Nguyen et al. [27] observed no change in overall youth cannabis use but noted increased initiation among new users, suggesting a need for additional measures to curb initiation. Doran et al. [22] reported stable marijuana use frequency post-legalization in California, with notable sex differences, with men reporting decreasing and women increasing marijuana use frequency over time. These findings suggest that legalization does not inherently increase use, possibly due to existing age restrictions and pre-existing community attitudes that support or oppose such policies [11]. Overall, these studies indicate that while the immediate effects of legalization on youth consumption may be minimal, the long-term impact and evolving usage patterns warrant continuous monitoring. Understanding the role of sociopolitical contexts and normative strategies in shaping these outcomes is crucial to informing more effective and nuanced policy interventions.

The potential risks associated with increased cannabis use among adolescents following legalization, however, warrant a closer look. To address these challenges effectively, policymakers and public health officials should adopt a multifaceted approach. This approach should, for example, include robust public education campaigns aimed at informing adolescents about the risks associated with cannabis use and promoting healthy decision-making. Additionally, implementing stringent regulations on cannabis advertising and marketing targeted at youth can help prevent the normalization of cannabis use. Furthermore, investing in prevention and intervention programs tailored to the needs of adolescents, particularly those from vulnerable communities, could be essential to reducing cannabis-related harm and promoting overall well-being.

While the studies presented offer valuable insights into the relationship between cannabis policy and adolescent use, it is essential to acknowledge their inherent limitations. One notable limitation is the reliance on self-reported data, which can be susceptible to recall bias or social desirability bias [41], potentially leading to inaccuracies in reporting cannabis use behaviors. Additionally, the use of cross-sectional designs in some studies limits the ability to establish causality and may introduce selection bias [42].

Furthermore, the generalizability of findings may be constrained by the characteristics of the study population and the geographical context in which the research was conducted. Studies focused on specific regions or demographic groups may not be representative of broader populations, thereby limiting the applicability of their findings [43].

Moreover, the dynamic nature of cannabis policy presents challenges in assessing its long-term effects on adolescent use [44]. Many studies may only capture short-term impacts, providing an incomplete understanding of the broader implications of cannabis policy changes.

In conclusion, trends in adolescent cannabis use following policy changes present a complex picture that requires a multifaceted policy approach. By taking into account public health implications, socioeconomic factors, age-specific needs, and the nuanced effects of decriminalization, policymakers can develop strategies that not only address the immediate impacts of cannabis use, but also contribute to the long-term sustainability of society. This balanced approach is crucial to navigating the twists and turns of cannabis policy in a way that promotes public health, social equity, and sustainable development.

## 5. Conclusions

Our review of the literature on the effect of cannabis policy changes on adolescent cannabis use draws attention to the need for careful consideration of the complex and varied nature of the phenomenon and its effects on the sustainable development of society. Local cultural attitudes, pre-existing levels of cannabis use, socioeconomic conditions, gender, age, and type of policy change are all factors influencing this relationship and need to be taken into account when developing and implementing such policies.

It is crucial to implement targeted interventions to address socioeconomic inequalities. This involves establishing programs aimed at reducing these disparities, such as providing affordable education, job training, and economic opportunities for disadvantaged youth. Additionally, it is important to incorporate provisions in cannabis licensing and regulations that promote diversity and inclusion in the industry. For instance, prioritizing licenses for individuals from communities that have been disproportionately affected by drug enforcement policies.

Moreover, when tailoring policies to diverse youth demographics, thorough research is essential. This includes understanding the specific needs and preferences of different youth populations, taking into account factors such as race, ethnicity, gender, sexual orientation, and socioeconomic status. Based on this research, prevention and intervention programs can be developed that are sensitive to cultural differences and community norms, ensuring they resonate with diverse youth.

Lastly, it is important to anticipate how society will respond to policy changes. Before implementing any changes to cannabis policy, thorough impact assessments should be conducted to understand potential consequences. Proactive communication with the public should also be undertaken, explaining the reasons behind the changes, the benefits they may bring, and any challenges anticipated. This could help ensure that policy changes are well-received and understood by the community.

This review reveals the importance of adopting a holistic and proactive approach to cannabis policy in order to achieve the goals of a sustainable society. Policymakers need to take into account the sometimes-conflicting interests and needs of different segments of the population, balancing short- and long-term benefits and risks when implementing these policies. In addition to taking public health into account, this means adopting measures to combat socioeconomic inequalities, adapting policies to the specific needs of young people by taking their diversity into account, and anticipating the consequences of policy changes in the social representations of young people. Only a balanced, evidence-based approach will enable cannabis policy changes to contribute to the well-being and sustainable development of society as a whole, and particularly of young people, who are the most vulnerable to these policy changes.

The significance of this review lies in its contribution to evidence-based policymaking. It establishes a basis for research and helps shape practical approaches to regulating cannabis in order to maintain sustainability across different areas such as policy making and decision making. By understanding the intricate relationship between changes in cannabis policy and adolescent usage patterns, policymakers can develop regulations that balance public health concerns with societal needs. For instance, they can use our findings to tailor prevention strategies, allocate resources effectively, and implement regulations that minimize potential harms associated with adolescent cannabis use.

However, there are still challenges and unanswered questions that require additional research. We need to understand the long-term effects of evolving cannabis policies on youth substance use and to develop targeted prevention strategies. While existing studies provide insights into short-term outcomes following policy implementation, understanding the enduring impacts is crucial for developing effective interventions and policies. Furthermore, it is crucial to develop targeted prevention strategies tailored to the specific needs of adolescents. Current interventions may not adequately address the diverse factors influencing adolescent cannabis use, highlighting the need for innovative approaches informed by rigorous research. To deepen our understanding of the consequences of cannabis policy changes, it is also crucial to explore how external events, such as the COVID-19 pandemic, impact adolescent cannabis consumption. The pandemic has disrupted social norms and access to resources, potentially influencing substance use behaviors among young people. Investigating these dynamics can provide valuable insights into emerging trends and inform responsive interventions. Addressing these gaps through rigorous research efforts is vital for advancing understanding and developing evidence-based approaches to mitigate the challenges posed by adolescent cannabis use. Prioritizing these areas for future research could better support the health and well-being of young people in the face of evolving cannabis policies and external stressors.

Additionally, our review underscores the importance of ongoing research and evaluation to continuously inform policy adjustments. As societal attitudes toward cannabis change and new challenges arise, it is crucial to adapt regulatory frameworks to align with the evolving needs and circumstances of society. Continuous research efforts can help identify emerging trends, assess the effectiveness of interventions, and guide policy refinements to address the ever-changing landscape of cannabis use among adolescents.

## Figures and Tables

**Table 1:** Summary of studies describing correlation between legislative drug policy changes and adolescent cannabis consumption.

No	First Author (Study Countries)	Year(s) of Data Collection	Year of Publication	Sample Characteristics	Source of Data	Type of Legislative Change	Findings	Explanation
1	Bae and Kerr [10] (USA, 48 states)	2008–2018	2020	Undergraduates aged 18–26 years attending college in US states that did (*n* = 234,669 in seven states) or did not (*n* = 599,605 in 41 states) enact RML between 2008 and 2018.	Cross-sectional National College Health Assessment Survey	Recreational cannabis legalization.	After adjusting for covariates and state-specific trends, the prevalence of 30-day cannabis use increased more among students exposed to RML, with an odds ratio of 1.23 (95% CI = 1.19–1.28, *p* < 0.001), and similar trends were observed for frequent use (≥20 days) with an odds ratio of 1.18 (95% CI = 1.10–1.27, *p* < 0.001).	Not specified.
2	Harpin et al. [11] (Colorado, USA)	2013–2014	2017	6th through 12th grade students 2013 (*n* = 12,240) and 2014 (*n* = 11,931) who were surveyed in 40 schools (19, 9th–12th grade high schools and 21, 6th–8th grade middle schools, some combined).	Healthy Kids Colorado Survey	Recreational cannabis legalization.	No significant changes were observed in the prevalence of ongoing use of cannabis, past 30-day use. Significant increase in perception of easy access to cannabis.	The persistence of cannabis use may be due to the prohibition for minors under 21 and the likelihood that many had already started using before legalization. Communities with more lenient views on cannabis may have higher use rates due to local ordinances and dispensaries, but behavior change could still occur with time despite media attention to cannabis policy.
3	Fischer et al. [12] (Canada)	2017–2020	2021	NCS: Sample size (*n*) = 12,000, Age range: 15 and older. CCS: Sample size (*n*) > 10,000, Age range: 16 and older. CSTADS: Targeted at grades 7–12, mostly ages 13–18. CAMH Monitor: Focus on adults aged 18 and over in Ontario. ICPS ^1^: Non-probability sample, ages 16–65.	National Cannabis Survey (NCS) Canadian Cannabis Survey (CCS) Canadian Student Tobacco, Alcohol, and Drugs Survey (CSTADS) The Centre for Addiction and Mental Health (CAMH) International Cannabis Policy Study (ICPS) Ontario Cannabis Store (OCS)	Recreational cannabis legalization.	NCS: the overall prevalence of cannabis use in the past three months increased from 14.9% in 2018 to 20.0% in 2020, with the highest prevalence among those aged 18–24 years. Among respondents aged 15–17, cannabis use fluctuated but did not show significant differences over this period. CCHS: cannabis use in the past 12 months increased from 21.9% in 2018 to 26.9% in 2020, with the age group of 20–24 years exhibiting the highest use. Cannabis use also increased among those aged 16–19 years during this time frame. CSTADS: cannabis use remained steady from 2016/17 to 2018/19, with an increase observed among younger students in grades 7–9 but no change in older students in grades 10–12. All age groups showed increasing trends in cannabis use between 2017 and 2019.	The rise in cannabis consumption has been linked to the COVID-19 pandemic.
4	Rotermann [7] (Canada)	To update long-term trends in 12-month cannabis prevalence from 2004 to 2017. To analyze cannabis consumption patterns using data collected every three months from early 2018 to 2019.	2019	CTUMS: (*n*) = 19,822 to 21,976 CTADS: (*n*) = 14,565 to 16,349 NCS (Quarterly): (*n*) = Averaged 5811 respondents.	Canadian Tobacco, Alcohol and Drugs Survey (CTADS) Canadian Tobacco Use Monitoring Survey (CTUMS) National Cannabis Survey (NCS)	Recreational cannabis legalization.	Between 2004 and 2017, the use of cannabis declined among 15 to 17-year-olds, while it remained consistent among 18 to 24-year-olds. From 2018 to 2019, the frequency of cannabis use in the last three months was notably greater among individuals aged 18 to 24 standing at 34.8%, surpassing the rates observed in other age brackets, ranging from 4% to 24%.	Continual alterations in policies regarding the legalization, regulation, and constraints on non-medical cannabis, alongside the evolving societal acceptance of its use, persist in influencing use trends.
5	Mæland et al. [13] (Norway)	2017–2019	2022	Norwegian adolescents from 2017 to 2019, covering about 80% of lower secondary school pupils (aged 13–15) and 50% of upper secondary pupils (aged 16–19). (*n*) = 249,100	Norwegian youth survey Ungdata	Depenalization (reduction of the severity of the penalties).	As age increased, so did cannabis consumption from 1.5% (1st year of lower secondary) peaking at 18.9% in the final year of upper secondary education. Additionally, a clear correlation emerged between lower family income and higher cannabis use, with figures ranging from 7.3% in consistently well-off families to 24.0% in financially struggling households.	Age and socioeconomic status.
6	Benedetti et al. [5] (20 European countries: Croatia, Czech Rep., Denmark, Finland, France, Greece, Hungary, Iceland, Italy, Latvia, Malta, Netherlands, Norway, Poland, Portugal, Romania, Slovak Rep., Slovenia, Sweden, Ukraine) (13 countries where laws were changed and 7 countries that served as a control group)	1999–2015	2021	Students who turn 16 years of age in the given survey year. Croatia (*n*) = 14,667 Czech Rep. (*n*) = 16,876 Denmark (*n*) = 8512 Finland (*n*) = 18,881 France (*n*) = 12,214 Greece (*n*) = 15,834 Hungary (*n*) = 14,151 Iceland (*n*) = 14,112 Italy (*n*) = 26,665 Latvia (*n*) = 10,852 Malta (*n*) = 17,035 Netherlands (*n*) = 10,397 Norway (*n*) = 15,294 Poland (*n*) = 28,521 Portugal (*n*) = 14,616 Romania (*n*) = 14,747 Slovak Rep. (*n*) = 10,971 Slovenia (*n*) = 14,550 Sweden (*n*) = 14,399 Ukraine (*n*) = 13,590	European School Survey Project (ESPAD)	Decriminalization (change in the status of cannabis use from a criminal to a non-criminal offence), depenalization (reduction of the severity of the penalties) and increase of the penalties (either civil or criminal).	Significant changes in the prevalence of cannabis use are associated with only two specific policy changes: depenalization through minor case closure facilitation of the closure of minor cases leads to an increase (6.6 percentage points) in cannabis use, while an increase in non-prison penalties results in a decrease (3.3 percentage points). These associations are consistent across all users, but no significant effects were observed for frequent cannabis users.	Certain reforms in cannabis policies were linked to notable shifts in prevalence.
7	Wieczorek et al. [4] (Poland’s results were compared to those obtained in specific European countries, namely the Czech Republic, France, Finland, and Ukraine.)	1995, 1999, 2003, 2007, 2011 and 2015.	2018	15- to 16-year-olds (*n*) Poland 2015 = 11,822 (*n*) Czech Republic 2015 = 2738 (*n*) Finland 2015 = 4049 (*n*) France 2015 = 2714 (*n*) Ukraine 2015 = 2350	European School Survey Project on Alcohol and Drugs (ESPAD)	Reduction of the severity of the penalties.	Between 1995 and 2015, experimental cannabis use among 15- to 16-year-olds in Poland rose threefold with 25% having tried it by 2015. Nearly 20% were occasional users, and close to 10% used it in the last 30 days before the study. Compared to other countries, Poland saw a significant increase while most others showed a decline in cannabis use, except for the Czech Republic, where the decrease began in 2007.	Individual psychosocial factors such as familiarity with drug sources and influence from peers and family members who use drugs seem to have a more substantial impact on drug prevalence compared to legal regulations governing drug supply and demand.
8	Nguyen and Mital [14] (Canada)	2018–2020	2022	15–20 years old (*n*) = 1005	National Cannabis Surveys	Increase in minimum legal age for recreational cannabis use in Quebec.	Despite the policy change, cannabis consumption did rise; however, the escalation in cannabis use among 18- to 20-year-olds was 51% less in Quebec compared to other provinces, while there was no noticeable shift in cannabis use among 15- to 17-year-olds.	The data indicate that while some youth may have resorted to the illegal market, the decrease in legal cannabis use outweighed the increase in illegal consumption.
9	Wang et al. [15] (USA)	2014–2015	2019	College students 18< median age was 24 years. (*n*) = 7105	Researched Abuse, Diversion and Addiction-Related Surveillance (RADARS)	Medical cannabis legalization. Recreational cannabis legalization. Non-legal states.	In states where recreational cannabis is permitted, 28% of college students have reported recent cannabis use, exceeding the rates observed in both non-legalized states (22%) and states with medical cannabis legalization (25%). The difference between states with medical cannabis legalization and those without legalization is statistically significant (*p* < 0.001). The likelihood of cannabis use is higher in both medical (OR: 1.23, 95% CI: 1.09–1.38) and recreational (OR: 1.46, 95% CI: 1.07–1.97) legalized states compared to non-legalized states. Additionally, students residing in states with medical cannabis legalization show a greater inclination for cannabis use compared to their counterparts in non-legalized states.	Decline in risk perception and the shift in public opinion are substantial factors contributing to the increased use of cannabis.
10	Smyth et al. [6] (Portugal, UK, Italy, Slovakia, Bulgaria, Finland, Poland, Denmark.)	1995–2017	2023	15–16-year-old school children (*n*) = 700,000 students	European School Survey Project on Alcohol and Drugs (ESPAD)	Penalty reduction. Penalty increase.	In eight out of ten cases analyzed, changes in penalties for cannabis use were aligned with deterrence theory, suggesting that stricter penalties were associated with a decrease in cannabis prevalence while more lenient penalties were correlated with increased use. The likelihood of these patterns occurring by chance was calculated at 0.05. The median change in prevalence around policy shifts was 21%, with instances of both increases and decreases in cannabis use observed.	Reducing penalties could potentially lead to slight upticks in adolescent cannabis use and, as a result, elevate the associated risks and harms linked to cannabis.
11	Mauro et al. [16] (USA)	2004–2013	2019	12–17 years old (*n*) = 17,500 18–25 years old (*n*) = 17,500 26 and older (*n*) = 18,000	National Survey on Drug Use and Health (NSDUH)	Medical cannabis legalization.	MML enactment did not impact cannabis use among 12–17-year-olds. For 18–25-year-olds, MML did not affect past-month use, but daily use increased notably among men after MML enactment.	MMLs might affect cannabis availability more for men, possibly as an alcohol substitute. Additionally, more men might use cannabis medicinally or to replace opioid use in this age group.
12	Schmidt et al. [17] (USA)		2019	12–25 years old (*n*) = 450,300	National Survey on Drug Use and Health (NSDUH)	Medical cannabis legalization.	Living in a state where medical cannabis was legalized did not correlate with recent cannabis use among early adolescents, late adolescents, or young adults. However, young adults in these states were notably more prone to starting cannabis use within the past year.	Adolescents at high-school age exhibit a unique susceptibility to social cues, potentially leading to increased experimentation with drugs. Moreover, in states where enforcement of medical cannabis regulations is comparatively lenient, young adults might demonstrate greater willingness to try cannabis due to their perception of reduced arrest risk or a generally diminished perception of the drug’s risks.
13	Rusby et al. [18] (USA)	2014–2015	2017	Average age 14.4 (*n*) = 444 Parents (age not specify) (*n*) = 343	Online questionnaires	Recreational cannabis legalization.	In places where sales were restricted, new users were less likely to start with cannabis, but in areas where it was legalized, more non-users expressed an interest. For existing young users, legalization correlated with increased use. Communities banning sales saw a higher increase in cannabis use among young users. Essentially, recreational cannabis legalization did not prompt new users, but it increased consumption among existing users. Legalization did not have an effect on parental use.	The prevailing rejection of recreational cannabis legalization in communities where its sale is banned by policy likely had an impact on the attitudes of young individuals, influenced by these community norms.
14	Bailey et al. [19] (USA)	2002–2011 2015–2018	2020	10–20 years old (*n*) = 281 youth	Seattle Social Development Project—The Intergenerational Project (SSDP-TIP)	Recreational cannabis legalization.	The legalization of nonmedical cannabis was associated with a notably increased chance of self-reported past-year cannabis use (AOR = 6.85, *p* = 0.001) and alcohol consumption (AOR 3.38, *p* = 0.034) among young individuals. However, this legalization did not show a significant link to past-year cigarette use (AOR = 2.43, *p* = 0.279) or a lower perception of harm from cannabis use (AOR = 1.50, *p* = 0.236) among youth aged 10 to 20 years.	After nonmedical legalization, there is still a connection between lower perceived harm and increased adolescent cannabis use.
15	Cerdá et al. [20] (USA)	1991–2015	2017	8th, 10th, and 12th graders (*n*) = 1,179,372 8th graders (*n*) = 423,899 10th graders (*n*) = 386,596 12th graders (*n*) = 368,877	Monitoring the Future (MTF)	Medical cannabis legalization.	The effects of enacting MML varied across different grades. After its enactment, 8th graders showed decreased use of cannabis. For 10th graders, there was no noticeable impact on substance use following MML implementation. However, among 12th graders, the use of cannabis remained largely unchanged.	When laws permitting medical cannabis are enacted, there might be more public communication regarding the dangers associated with adolescents using cannabis. Eighth graders may be more affected by these cautionary messages compared to other age groups, particularly in relation to cannabis use. Furthermore, parents of younger adolescents might be more inclined to monitor their children’s substance use after these laws come into effect. Research suggests that parental impact is most significant during early adolescence but tends to decrease as adolescents mature.
16	Doggett et al. [21] (Canada)	2017–2019	2022	Grades 9–12 (*n*) = 18,824	Comparative Policy Analysis for Sustainable Societies (COMPASS)	Recreational cannabis legalization.	Over the course of the study, there was a noticeable inclination among youth to increase the frequency of their cannabis consumption. Moreover, individuals predominantly engaged in cannabis smoking at the initial assessment were observed to transition towards employing multiple modes of cannabis consumption.	It is widely acknowledged that as young individuals age, there is a natural inclination towards engaging in substance-use behaviors. This is compounded by the prevailing understanding from research suggesting a lack of awareness among youth regarding cannabis edibles. Furthermore, young people express a preference for alternative modes of cannabis consumption due to a perception of these methods as being “healthier” choices.
17	Doran et al. [22] (USA)	2015–2016	2020	18–24 years old (*n*) = 563	Longitudinal study	Recreational cannabis legalization.	The frequency of cannabis use remained consistent over time, even after legalization. More frequent use was linked to younger age and self-identification as white (*p* < 0.001), a trend that persisted post-legalization. The frequency of cannabis use was influenced by gender (*p* < 0.001), indicating an increase in use among women and a decrease among men as time progressed.	Females seem to have a heightened responsiveness to the pleasurable effects of cannabis consumption, potentially making them more susceptible to increased use post-initiation or in situations where obstacles to use are decreased.
18	Zuckermann et al. [23] (Canada)	2012–2013 2017–2018	2022	Grade 9–12 students (*n*) = 230,404	Comparative Policy Analysis for Sustainable Societies (COMPASS)	Recreational cannabis legalization.	Following a consistent decline in youth cannabis use over the span of several years, there seems to have been a gradual upturn in cannabis consumption among this demographic, particularly in the wake of discussions regarding cannabis legalization. This increase poses elevated risks for certain subsets of the youth population.	In Canada, indicators suggest a normalization of cannabis use, likely due to increased accessibility. This is notably true for young people, especially female students, whose historically stigmatized cannabis use has been affected more. With legalization, access to and normalization of diverse cannabis products are expected to rise.
19	Evans et al. [24] (USA)	2017	2020	18< (*n*) = 3022	Cross-sectional mail and web-based survey.	Recreational cannabis legalization.	In Massachusetts, a study revealed that men were more inclined than women to use cannabis, as were individuals aged 18 to 20 in comparison to those aged 21 to 25.	The increased reported use of cannabis in Massachusetts could be due to evolving public opinions and changes in laws and policies related to cannabis, potentially influencing adolescents’ perception of risk and increasing their likelihood of use.
20	Hammond et al. [25] (Canada, England, USA)	2017–2019	2021	16- to 19-year-old Canada (*n*) = 11,779 England (*n*) = 11,117 USA (*n*) = 11,869	Cross-sectional online surveys.	Recreational cannabis legalization. Medical cannabis legalization. Recreational cannabis legalization.	The use of cannabis was more common in Canada and the US compared to England across all years and saw a more substantial increase between 2017 and 2019 (*p* < 0.001 for all comparisons). Specifically among those who had used cannabis in the past 30 days, the prevalence of vaping oils/liquids and the use of cannabis extracts (oil, wax, and shatter) rose in all countries, with notably higher rates in Canada and the US.	The study found mixed evidence regarding the impact of cannabis legalization on youth. While cannabis use was more prevalent in places where medical cannabis is legal, these differences primarily reflected pre-existing trends. The impact of recreational cannabis legalization appeared mixed, partly due to the recent nature of these changes and the time required to establish legal retail markets.
21	Mennis et al. [26] (USA)	2008–2019	2022	12–25 (*n*) = 1155	National Survey of Drug Use and Health (NSDUH) Treatment Episode Dataset—Admissions (TEDS-A)	Recreational cannabis legalization.	Following the legalization of recreational cannabis, there was an increase in the prevalence of past-month cannabis use among adolescents and young adults;	A stronger connection was observed between lower perception of the risk of harm and higher prevalence of cannabis use among both adolescents and young adults.
22	Nguyen et al. [27] (Canada)	CTUMS 1999–2012 CTADS and CTNS 2013–2017 and 2018	2022	15 years and older CTUMS (*n*) = 20,000 CTADS (*n*) = 15,000 CTNS (*n*) = 8500 grades 6–12 YSS (*n*) = 40,000	Canadian Tobacco Use Monitoring Surveys (CTUMS) Canadian Tobacco Alcohol & Drugs Surveys (CTADS) Canadian Tobacco & Nicotine Survey (CTNS) Youth Smoking Survey (YSS)	Recreational cannabis legalization.	While the overall prevalence of youth cannabis use remained unchanged, there was a 69% increase in cannabis initiation following legalization.	The environmental and social settings impact how young individuals view the risks of cannabis and whether they decide to use it. After legalization, there was a delay in the number of years before youth started using cannabis. Additionally, while legalization made cannabis more accessible to young people, it also heightened their perception of its potential harm.
23	Stormshak et al. [28] (USA)	2000–2010 2009–2018	2019	Early high school—24 (*n*) = 1468	Two longitudinal projects.	Recreational cannabis legalization.	The findings show increased cannabis use among individuals in Sample 2 during their young adult years, coinciding with the introduction of RML in Oregon. Young adults in Sample 2 had 2.12 times higher odds of using cannabis at age 24 compared to those in Sample 1, and they reported more frequent use across multiple time points in young adulthood. Overall, these results suggest that young adults after RML are more likely to use cannabis compared to their counterparts a decade earlier.	Not specified.
24	Vaičiūnas et al. [29] (Estonia, Latvia, Lithuania, Poland)	1994–2018	2022	15 years old (*n*) = 42,169	Health Behaviour in School-aged Children survey	Penalty reduction.	Over the past decade, cannabis use within the 30-day period varied in the Baltic states and Poland. Among males, it fluctuated more (5% to 13%) than among females (2% to 8%). The Baltic states saw an increase in cannabis use (TJT = 38.50, z = 1.651, *p* = 0.099), while Poland remained stable, showing a sharper decline after 2014 (4–8%, TJT = 4.50, z = 1.083, *p* = 0.279), albeit not a statistically significant one. From 1994 to 2002, the Baltic states showed a general increase, followed by stability between 2002 and 2010, then declining trends from 2010. Poland had less consistent patterns, with declining trends beginning earlier. The prevalence of cannabis use, measured since 2006, displayed unique fluctuations within and among countries.	The use of cannabis suggests insufficient or ineffective national-level cannabis control policies and enforcement, contributing to its increasing popularity in certain countries, such as Lithuania.

^1^ We did not include data from ICPS in our paper results due to its lack of age-group differentiation.

**Table 2:** Prevalence of cannabis use following policy changes.

Type of Policy Changes	Increase	Decrease	No Change
Recreational cannabis legalization	Bae and Kerr [10] Fischer et al. [12] Rotermann [7] Rusby et al. [18] Bailey et al. [19] Doggett et al. [21] Zuckermann et al. [23] Evans et al. [24] Mennis et al. [26] Stormshak et al. [28]		Harpin et al. [11] Doran et al. [22] Nguyen et al. [27]
Depenalization	Mæland et al. [13]		
Decriminalization, depenalization, increase in penalties	Benedetti et al. [5]	Benedetti et al. [5]	
Reduction of the severity of the penalties (penalty reduction)	Wieczorek et al. [4] Vaičiūnas et al. [29]	Wieczorek et al. [4] Vaičiūnas et al. [29]	Vaičiūnas et al. [29]
Increase in minimum legal age for recreational cannabis use	Nguyen and Mital [14]		
Legalization for medical use, Legalization for recreational use, non-legal states	Wang et al. [15]		
Penalty reduction, penalty increase	Smyth et al. [6]	Smyth et al. [6]	
Medical cannabis legalization	Mauro et al. [16] Cerdá et al. [20]	Cerdá et al. [20]	Mauro et al. [16] Schmidt et al. [17] Cerdá et al. [20]
Recreational cannabis legalization, medical cannabis legalization	Hammond et al. [25]		

## Data Availability

The data underlying this study’s results are available upon request.
